# The Idle Moments of Busy Men

**Published:** 1905-08

**Authors:** F. C. Brush

**Affiliations:** New York, N. Y.


### THE IDLE MOMENTS OF BUSY MEN.[1]

BY DR. F. C. BRUSH, NEW YORK, N. Y.

THE thought often comes to young men, How is it that busy men find the time for study, research, preparation of professional papers, devising of instruments and methods, and the kindred other things that we have continual evidence they are doing?

It is really the busiest man that has the most time for varied things, for he is one that has learned to make use of every fleeting moment. He has few idle moments, yet many for rest and recreation, as true rest is not idleness but a change; even a change of thought, not necessarily a decided change of occupation. The developing of a new thought or the pursuit of some investigation is a mental rest and recreation that produces its good result quite as well as the more violent physical exercises which so many deem essential. To obtain the best results from this kind of a mental change, one must, of course, have a great love for one's work and all its details; so much so that it becomes a hobby rather than the

---

[1] Read before The New York Institute of Stomatology, April 4, 1905.

mere daily drudgery which must be pursued for its financial returns. It is the kind of love that takes possession of a man and causes him, when his office hours are drawing to a close, to long for the restful hours of evening, when he may develop some thought or work out some problem that has come to him during the routine work of the day; and then when the evening is waning and another day approaching, to long for the morning, that he may return to the office and put into execution and test the results of the evening's labor.

Some time since I had the good fortune to gain the confidence and friendship of such a man, and with it an opportunity to know him more intimately. As I learned to know and understand him, and to see more of his habits and methods, he became a constant revelation and inspiration to me. I began to understand something of his success, aside from the charm of his personality and intense individuality. Many little traits and habits that at first were inexplicable to me were understood later, and their meaning became clear. In the midst of the proceedings of a meeting I have observed him hastily writing on some scrap of paper the gist of a telling point made by a speaker, a bit of new information or a thought of his own that may have come to him on the subject; later, when he has shown a surprising fund of knowledge on a wide range of subjects, I have realized how a deal of this knowledge has been absorbed and laid away for use at a fitting time. Though knowing him to be a linguist of ability, I was surprised on one occasion to hear him make use of still another language; upon inquiry, he informed me that for some time it had been his habit to have a grammar of this language upon his dresser and to learn a certain number of the words each morning while arranging his toilet.

How many of we younger men would have the courage and tenacity of purpose to undertake a task like that? And yet we sit with idle hands and wonder how they do it—we haven't the time. To be sure, we cannot all be "Bogues," yet how many there are that are idling and complaining they haven't time, who, if they would but apply themselves systematically, would find that measure of success which all are seeking. It is surprising how much time we may find we have, and the things we may accomplish, if we will but get busy and utilize the spare moments.

How can we do this? Well, a good plan is to have a memorandum pad handy by the operating-chair, and when, in the midst of an operation, an idea comes regarding the work in hand, or of an instrument or something that may be arranged to facilitate your work or add to the comfort of the patient, jot it down. These little notes should be filed in a conspicuous place on your desk, and when an opportunity comes, such as a rainy day or a broken appointment, you may turn to them and find at once something you can be doing, instead of sitting around and rummaging your brain in a futile effort to recall that very important thing you were going to do the very first chance you had.

If while reading the journals or engaged in your work a good idea comes to you on the subject, try to write it out and express it in a way to be clearly understood by others who may not be in touch with your own train of thought. Then when your society calls upon you to do your part, you will have something to turn to, and from your notes you may no doubt be enabled to evolve a paper which will be creditable to yourself and of some material help to others. If such prove to be the case, the notes will not have been in vain nor the time wasted.

Another excellent plan is to observe closely the mouths of all patients that come under our care. Not the mere searching out of cavities, that fillings may be inserted and fees obtained, but observing all conditions carefully, that we may study them and compare and reflect.

We labor under the same general disadvantage as does the physician, in that we are called upon to treat the results of pathological conditions rather than normal ones. Rarely is it that we have an opportunity of examining a mouth that is perfectly normal, particularly in regard to the form, shape, and size of the arches, the shape of the teeth, their position in the alveolus, and their relation one with another. Unless we have a clear idea of what the normal is or should be, how can we pass judgment on these abnormal or pathological conditions and feel that the treatment purposed is the best for the case in hand?

Have we gone beyond the study of the best filling-material for some individual tooth, and considered the important part which that tooth plays in the economy of mastication and vocalization, not merely as a crusher of food or as an aid in the enunciation of certain sounds, but as an integral part of the whole machine, that has an essential function to perform in preserving and regulating the harmony of all the other parts? How can we appreciate all this if our attention is continually centred only upon the small part which is our immediate field of operation?

As an aid in studying this broader field, I try, whenever possible, to secure models of mouths that present what we may call unusual conditions, whatever they may be. These models are being filed away for future reference, study, and comparison, and they have proved of inestimable value in diagnosing and prognosing other cases.

It may be permissible in this connection to relate an incident that occurred in my practice a short time ago. A patient, while in the operating-chair, volunteered the information that the young lady accompanying her had beautiful teeth, and had never required the services of a dentist. I stated that if such were the case there must certainly be a good reason for it, and I believed that reason to be that nature had provided her with a practically normal mouth, and I dared say I could describe the general conditions of her mouth, the positions of the teeth, and their relation one to another. The patient became interested, and called her friend into the operating-room. An examination was made, and, to the surprise of the young ladies and my gratification, it was found that my description was essentially correct. This incident made quite an impression on the young ladies, and I feel that there are at least two more people who have a higher appreciation of the importance of saving their teeth and, I trust, of the services that dentistry aims to render.

But—some of you may say—we are busy men, and haven't the time for such things. That no doubt is true, but it is the truly busy man that in his idle moments finds the time for many things.

